# Workflow Intervals and Outcomes of Endovascular Treatment for Acute Large-Vessel Occlusion During On-Vs. Off-hours in China: The ANGEL-ACT Registry

**DOI:** 10.3389/fneur.2021.771803

**Published:** 2021-12-21

**Authors:** Yunlong Ding, Feng Gao, Yong Ji, Tingting Zhai, Xu Tong, Baixue Jia, Jian Wu, Jiaqi Wu, Yanrong Zhang, Can Wei, Wenjuan Wang, Jue Zhou, Jiali Niu, Zhongrong Miao, Yan Liu

**Affiliations:** ^1^Department of Neurology, JingJiang People's Hospital, The Seventh Affiliated Hospital of Yangzhou University, Taizhou, China; ^2^Stroke Center, JingJiang People's Hospital, The Seventh Affiliated Hospital of Yangzhou University, Taizhou, China; ^3^Hospital Office, JingJiang People's Hospital, The Seventh Affiliated Hospital of Yangzhou University, Taizhou, China; ^4^Department of Interventional Neuroradiology, Beijing Tiantan Hospital, Capital Medical University, Beijing, China; ^5^Department of Rehabilitation, JingJiang People's Hospital, The Seventh Affiliated Hospital of Yangzhou University, Taizhou, China; ^6^Department of Clinical Pharmacy, JingJiang People's Hospital, The Seventh Affiliated Hospital of Yangzhou University, Taizhou, China

**Keywords:** endovascular treatment, on-hours, off-hours, acute ischemic stroke, large vessel occlusion

## Abstract

**Background:** There may be a delay in or a poor outcome of endovascular treatment (EVT) among acute ischemic stroke (AIS) patients with large-vessel occlusion (LVO) during off-hours. By using a prospective, nationwide registry, we compared the workflow intervals and radiological/clinical outcomes between patients with acute LVO treated with EVT presenting during off- and on-hours.

**Methods:** We analyzed prospectively collected Endovascular Treatment Key Technique and Emergency Work Flow Improvement of Acute Ischemic Stroke (ANGEL-ACT) data. Patients presenting during off-hours were defined as those presenting to the emergency department from Monday to Friday between 17:30 and 08:00, on weekends (from 17:30 on Friday to 08:00 on Monday), and on national holidays. We used logistic regression models with adjustment for potential confounders to determine independent associations between the time of presentation and outcomes.

**Results:** Among 1,788 patients, 1,079 (60.3%) presented during off-hours. The median onset-to-door time and onset-to-reperfusion time were significantly longer during off-hours than during on-hours (165 vs. 125 min, *P* = 0.002 and 410 vs. 392 min, *P* = 0.027). The rates of successful reperfusion and symptomatic intracranial hemorrhage were similar in both groups. The adjusted odds ratio (OR) for the 90-day modified Rankin Scale score was 0.892 [95% confidence interval (CI), 0.748–1.064]. The adjusted OR for the occurrence of functional independence was 0.892 (95% CI, 0.724–1.098), and the adjusted OR for mortality was 1.214 (95% CI, 0.919–1.603).

**Conclusions:** Off-hours presentation in the nationwide real-world registry was associated with a delay in the visit and reperfusion time of EVT in patients with AIS. However, this delay was not associated with worse functional outcomes or higher mortality rates.

**Clinical Trial Registration:** URL: https://www.clinicaltrials.gov; Unique identifier: NCT03370939.

## Introduction

Stroke is the leading cause of death and disability in China (1). Ischemic stroke accounts for 65% (2) of stroke patients in China, of whom 35–40% have large-vessel occlusion (LVO) (1). LVO results in a large ischemic area and can cause severe brain damage (3, 4), leading to high mortality and disability rates (5–7). As a landmark in the treatment of acute ischemic stroke (AIS) with proximal intracranial LVO (8–12), endovascular treatment (EVT) has been widely used in real-world clinical practice. However, stroke causes 2 million neurons to undergo apoptosis every minute, and the brain ages 3.6 years per hour (13). Therefore, performing EVT as soon as possible in patients with LVO is the key to improving patient prognosis (14, 15).

Approximately half of patients present during so-called off-hours, i.e., evenings, nights, weekends, and holidays, and EVT needs to be performed jointly by emergency department staff, digital subtraction angiography (DSA) room nurses, technicians, anesthesiologists, and surgeons. Therefore, off-hour presentation may be associated with a delay in the start of EVT. The MR CLEAN trial found that presenting off-hours was associated with a slight delay of EVT but that this treatment delay did not translate into worse functional outcomes or an increased rate of complications (16). Data on off-hours delays in workflow intervals have shown delays in different workflow intervals and related performance, but these data are mostly based on single-center studies and are difficult to interpret given variations in acute stroke care (17–20). However, there have been no multicenter studies on the association of off-hour presentation and EVT workflow intervals in Chinese patients with LVO.

The Endovascular Treatment Key Technique and Emergency Work Flow Improvement of Acute Ischemic Stroke (ANGEL-ACT) registry was established to evaluate the utilization and subsequent outcomes of AIS patients who received EVT and has confirmed that favorable outcomes of EVT can be achieved in clinical practice in China (21). In this study, we analyzed prospectively collected ANGEL-ACT data to observe whether the workflow intervals and radiological/clinical outcomes were different between patients with acute LVO treated with EVT who presented during on-vs. off-hours.

## Methods

### Study Participants

Data were derived from the ANGEL-ACT registry. ANGEL-ACT was a nationwide, prospective, observational study of 1,793 consecutive adult patients with acute LVO treated with EVT at 111 hospitals from 26 provinces in China between November 2017 and March 2019 (https://www.clinicaltrials.gov; unique identifier: NCT03370939). Detailed information about the ANGEL-ACT registry can be found in a previously published article (21). Ethics approval was granted by the ethics committees of Beijing Tiantan Hospital and all participating centers. Subjects or their representatives provided written informed consent.

For the present study, patients who adhered to the following criteria were included: (1) age ≥18 years; (2) diagnosis of AIS on computed tomography (CT) angiography confirming intracranial LVO; and (3) initiation of any type of EVT, including mechanical thrombectomy, intra-arterial thrombolysis, stenting and angioplasty. Patients were divided into the on-hours group and the off-hours group based on their presentation time.

### Data Collection and Outcome Measures

Off-hours presentation was defined as presentation to the emergency department (participating centers) from Monday to Friday between 17:30 and 08:00, on weekends (from 17:30 on Friday to 08:00 on Monday), and on national holidays.

All variables, including demographic data, medical history, vital signs, laboratory and neurovascular imaging results, workflow intervals, and clinical outcomes, were prospectively collected.

The workflow intervals included the door-to-puncture time, onset-to-puncture time, onset-to-door time, door-to-imaging time, onset-to-needle time, puncture-to-reperfusion time and onset-to-reperfusion time. The radiological and clinical outcomes included the 90-day modified Rankin Scale (mRS) score as an ordinal variable, functional independence (defined as a 90-day mRS score from 0 to 2), mortality within 90 days, successful reperfusion [defined as the modified Thrombolysis in Cerebral Infarction (mTICI) score of 2b or 3 (22)], and symptomatic intracranial hemorrhage (sICH) within 24 h according to the Heidelberg Bleeding Classification (23).

### Statistical Analysis

Statistical analyses were performed using SAS 9.4 (SAS Institute, Inc., Cary, NC). All data are described as the median [interquartile range (IQR)] for continuous/ordinal variables and number (percentage) for categorical variables. The Wilcoxon test was used for continuous/ordinal variables, and Fisher's exact test or the chi-square test was used for categorical variables. A *P*-value of < 0.05 was considered statistically significant. Multivariable logistic regression models were used to determine the independent associations between the time of presentation (on-vs. off-hours) and radiological/clinical outcomes with adjustment for age, pretreatment with intravenous thrombolysis (IVT), baseline NIHSS score, occlusion site, prestroke mRS score, and onset-to-door time.

## Results

### Baseline Characteristics

Among the 1,793 subjects included in the ANGEL-ACT registry, 5 were excluded because the admission time was missing; thus, 1,788 patients were eligible for analyses. In total, 1,079 patients (60.3%) presented to the emergency department during off-hours, and 709 (39.7%) presented during on-hours. The baseline characteristics were similar in both groups except for the proportion of transferred patients. More patients in the off-hours group than in the on-hours group were transferred from primary stroke centers (36.98 vs. 32.02%, *P* = 0.033) (Table 1).

**Table 1:** Baseline Characteristics (median, IQR/*n*, %).

**Items**	**On-hours (*n* = 709)**	**Off-hours (*n* = 1,079)**	***P***
Age, y	65 (55–73)	66 (56–73)	0.496
Men	469 (66.2)	705 (65.3)	0.760
Baseline NIHSS (*n* = 1,780)	16 (12–22)	16 (12–21)	0.320
PremRS (*n* = 1,787)	0 (0–0)	0 (0–0)	0.539
Baseline ASPECTS (*n* = 1,773)	9 (7–10)	9 (7–10)	0.414
SBP (mmHg)	145 (130–160)	145 (131–162)	0.882
**Medical history**			
Hypertension	411 (58.0)	616 (57.1)	0.732
Diabetes	118 (16.6)	213 (19.7)	0.106
Hyperlipidemia	66 (9.3)	93 (8.6)	0.612
Coronary heart disease	111 (15.7)	162 (15.0)	0.737
Atrial fibrillation	217 (30.6)	344 (31.9)	0.602
Previous stroke	159 (22.4)	238 (22.1)	0.862
Smoking (recent or current)	292 (41.2)	424 (39.3)	0.430
IVT performed	491 (69.3)	774 (71.7)	0.265
Interhospital transfer	227 (32.0)	399 (37.0)	0.033
**Anesthesia**			0.384
Local anesthesia only	322 (45.4)	455 (42.2)	
General anesthesia	274 (38.7)	447 (41.4)	
Local anesthesia plus sedation	113 (15.9)	177 (16.4)	
**Occlusion site**			0.912
Internal carotid artery	185 (26.1)	269 (24.9)	
M1	298 (42.0)	471 (43.7)	
Basilar/vertebral artery	149 (21.0)	222 (20.6)	
Other	77 (10.9)	117 (10.8)	
**Stroke classification**			0.875
Large atherosclerotic stroke	344 (48.5)	533 (49.4)	
Cardiogenic cerebral embolism	229 (32.3)	346 (32.0)	
Other stroke with definite etiology	79 (11.1)	124 (11.5)	
Stroke of unknown etiology	57 (8.0)	76 (7.0)	

*Total number is 1,788 unless otherwise indicated. NIHSS indicates National Institutes of Health Stroke Scale; mRS, modified Rankin Scale; ASPECTS, Alberta Stroke Program Early CT Score; SBP, systolic pressure; IVT, intravenous therapy; M1, first segment of the middle cerebral artery*.

### Workflow Intervals

The median onset-to-door time during off-hours presentation was 165 (IQR: 70–295) minutes, which was significantly longer than that during on-hours presentation [125 (IQR: 60–260) min, *P* = 0.002]. The median onset-to-reperfusion time was also significantly longer during off-hours [410 (IQR: 310–561) min vs. 392 (IQR: 285–546) min, *P* = 0.027]. The door-to-puncture time, onset-to-puncture time, door-to-imaging time, onset-to-needle time and puncture-to-reperfusion time were similar between the two groups (Table 2).

**Table 2:** Workflow intervals (median, IQR).

**Items**	**On-hours (*n* = 709)**	**Off-hours (*n* = 1,079)**	***P***
Door-to-puncture time (*n* = 1,787)	124.5 (81.5–190)	123 (83–175)	0.164
Onset-to-puncture time (*n* = 1,774)	290 (200–431)	305 (218–445)	0.078
Onset-to-door time (*n* = 1,754)	125 (60–260)	165 (70–295)	0.002
Door-to-imaging time (*n* = 1,523)	15 (0–30)	14 (0–28)	0.345
Onset-to-needle time (*n* = 410)	160 (110–220)	159.5 (119.5–213)	0.843
Puncture-to-reperfusion time (*n* = 1,788)	80 (50–128)	88 (55–130)	0.078
Onset-to-reperfusion time (*n* = 1,774)	392 (285–546)	410 (310–561)	0.027

### Radiological and Clinical Outcomes

**Figure 1:**
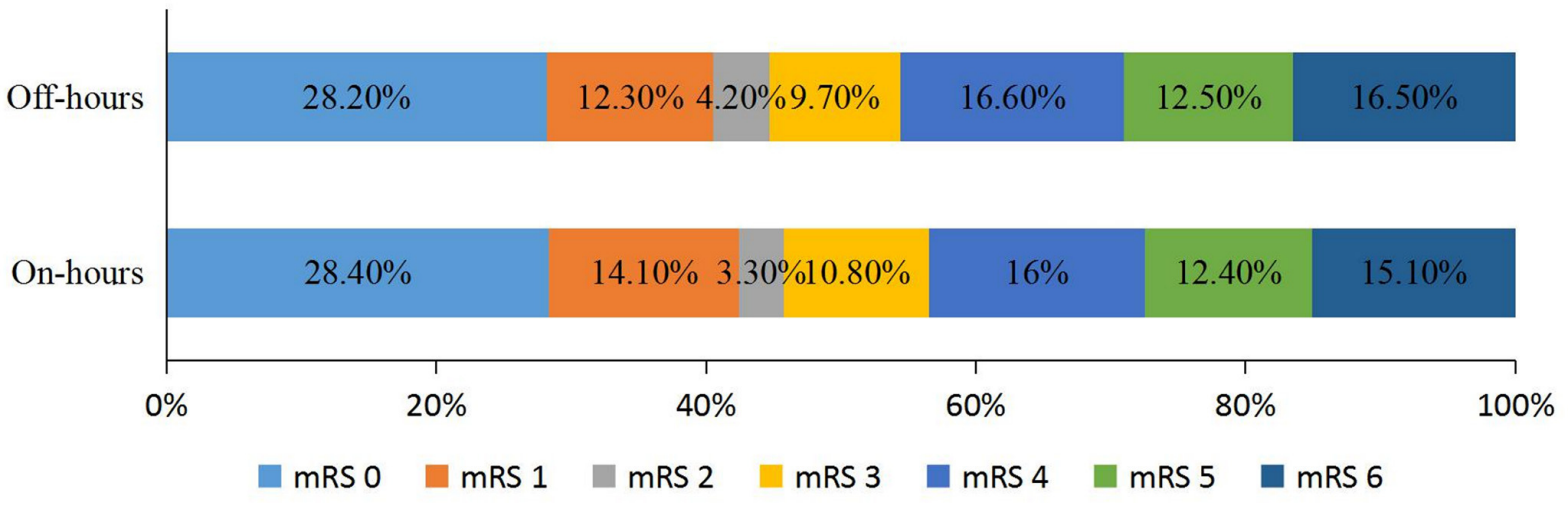
Distribution of modified Rankin Scale (mRS) scores at 90 days.

The rates of successful reperfusion and sICH were similar in both groups. The adjusted OR for the 90-day mRS score was 0.892 [95% confidence interval (CI), 0.748–1.064] (Table 3; Figure 1). The adjusted OR for the occurrence of functional independence was 0.892 (95% CI, 0.724–1.098), and the adjusted OR for mortality was 1.214 (95% CI, 0.919–1.603) (Table 3).

**Table 3:** Clinical outcomes (median, IQR/*n*, %).

**Items**	**On-hours (*n* = 709)**	**Off-hours (*n* = 1,079)**	**Unadjusted OR (95% CI)**	**Unadjusted *p*-value**	**Adjusted OR (95% CI)**	**Adjusted *p*-value**
mRS at 90 d (*n* = 1,771)	3 (0–5)	3 (0–5)	0.942 (0.794–1.118)	0.493	0.892 (0.748–1.064)	0.204
mRS (0–2) at 90 d (*n* = 1,771)	306 (45.7)	466 (44.7)	0.960 (0.790–1.166)	0.691	0.892 (0.724–1.098)	0.280
Reperfusion rate (TICI 2b−3) (*n* = 1,788)	618 (87.2)	955 (88.5)	1.134 (0.850–1.514)	0.414	1.087 (0.809–1.462)	0.579
Mortality at 90 d (*n* = 1,771)	101 (14.3)	172 (15.9)	1.142 (0.875–1.490)	0.347	1.214 (0.919–1.603)	0.172
sICH within 24 h (*n* = 1,695)	56 (8.4)	74 (7.2)	0.842 (0.586–1.209)	0.352	0.878 (0.606–1.272)	0.492

*mRS, modified Rankin Scale; TICI, Thrombolysis in Cerebral Infarction; sICH, symptomatic intracranial hemorrhage*.

## Discussion

This large-scale multicenter study reported the relationship of on- and off-hour presentation with workflow intervals and radiological/clinical outcomes and included over 1,700 patients treated at 111 different comprehensive stroke centers in China. This study showed that the visit and reperfusion times of EVT were 40 and 18 min later, respectively, in AIS patients presenting off-hours than in those presenting on-hours. There was no difference in the rates of successful reperfusion, sICH, or functional independence at 90 days, the mRS score distribution or mortality.

Similar to previous multicenter studies (16, 24–27), this study did not reveal a difference in the prognosis or rate of complications between the two groups. Benali et al. observed a significantly increased rate of good functional outcomes among inpatients admitted at night (51 vs. 35%, *P* = 0.05) (28), but another study found a higher mortality rate among patients admitted at night for EVT (29), which may be due to heterogeneity in stroke center processes and sample sizes. Although this multicenter study did not observe a difference in the effect of on-vs. off-hours presentation on the prognosis, exploring OR trends and differences in the workflow intervals between the two groups may help to improve the efficiency of EVT implementation.

Off-hours patients have a longer onset-to-door time. The 40-min gap suggests more delays before the hospitalization of off-hours patients. Experiencing a stroke after waking up at night may be an important contributing factor. Another factor may be transfers, as a higher proportion of off-hours patients required transfer. We hypothesize that some hospitals may not have the capability to provide EVT over 24 h, so patients who are first diagnosed at these hospitals during off-hours may need to be transferred to a suitable stroke center. Previous research has also described this phenomenon (16). Notably, in the treatment of patients requiring EVT, interhospital transfer will increase the onset-to-first door time (30–32). Therefore, when establishing the EVT process, the delay caused by referral should be recognized, special attention should be given to the impact of off-hours referrals, and effective public information campaigns should be used to make patients aware of hospitals with 24-h EVT capabilities in advance to ensure that patients can be delivered directly after stroke onset.

The overall door-to-puncture time in this study was 124 min, which is considerably greater than the 85 min reported in a prospective, randomized, controlled study conducted in China (33) and exceeds the requirement of 90 min for advanced Chinese stroke centers, suggesting that the EVT process needs to be further optimized in the real world. Given that door-to-imaging and door-to-needle times were relatively short, a better understanding of the reasons for the long door-to-puncture times would be helpful. Possible factors include but are not limited to the frequency of using advanced imaging modalities [i.e., computed tomography (CT) perfusion or magnetic resonance imaging (MRI)], some centers waiting for IVT finished prior to taking patients for EVT, and the proportion of patients who presented in the late window (i.e., >6 h from onset).

Our research showed that the median onset-to-reperfusion time in off-hours patients was 410 min, which is 18 min longer than that in on-hours patients. This finding indicates a certain delay in the EVT process for patients presenting off-hours. However, the time difference of 40 min in the onset to door times between the off-hours vs. on-hours patients was reduced to only 18 min of a difference in the onset to reperfusion times, suggesting more efficient in-hospital workflow during off-hours. We hypothesize that the possible reasons are as follows: (1) There were fewer patients in general during off-hours, a high proportion of idle machines and a reduced waiting time for patients. (2) More patients were interhospital transfers, and these patients may have received some examinations and learned about relevant treatments, reducing the time for communication after admission.

We found that the reperfusion rate in the off-hours group was similar to that in the on-hours group, whereas the puncture-to-reperfusion time in the off-hours group was 8 min longer than that in the on-hours group. We hypothesize that such a time difference may be attributed to the availability of the off-hours intervention team. Based on experience at our center, for patients who require general intravenous anesthesia, the response of the anesthesiologist during off-hours may not be as good as that during on-hours. Furthermore, more junior doctors may be on duty during off-hours, the interventionalists available off-hours may not be as experienced as those available on-hours, and physician or staff fatigue during late-night procedures may cloud judgment or increase the risk of procedural complications (34, 35). In the future, attention needs to be given to optimizing the configuration of the intervention team during off-hours to reduce the puncture-to-reperfusion time.

The definition of off-hours was set according to statutory holidays and time points. The ANGEL-ACT registry contains data from 111 hospitals in 26 provinces in China (21). These hospitals operated under Beijing time in Dongba District, which is the standard time in China. Therefore, it is possible that when Dongba District has entered the evening, the Eastern Fifth District may still be in daytime hours. Thus, it is necessary to carefully define off-hours. We analyzed the locations of the 111 hospitals and found that 106 (95.4%) hospitals were located in the time zone of the East 8th District and East 7th District. These hospitals accounted for 1716 (95.9%) patients. Thus, the time zone difference for 95.9% patients was <1 h. We conducted a survey on the work and rest time of all hospitals in the group. In total, 98.2% of the hospitals' work hours were 7:30–18:30 with off-hours of 17:00–8:00 in both summer and winter. Therefore, it is reasonable to choose the hours between 08:00 and 17:30 when defining the time points of off-hours.

This study has some limitations. First, 17 of 1,788 patients did not have 3-month follow-up data available from phone interviews; thus, the rates of poor outcomes or serious events may have been underestimated. Second, Saad et al. found that the workflow interval had no effect on EVT in teaching hospitals but did have an effect on EVT in non-teaching hospitals (36). Our study included stroke centers with 24-h EVT capability, and most of these centers were associated with teaching hospitals. Therefore, whether the results can be extended to all stroke centers in China remains unclear. Finally, our definition of off-hours included statutory holidays. However, some hospitals included in the study follow a normal work schedule on statutory holidays, and some hospitals even have a 24-h neurointervention emergency team on duty.

## Conclusion

In conclusion, according to the nationwide real-world registry, off-hour presentation was associated with a delay in the visit and reperfusion time of EVT in patients with AIS. However, this delay was not associated with worse functional outcomes or greater mortality. In future optimization of the EVT process during off-hours, the onset-to-door time and onset-to-reperfusion time should be key targets for improvement.

## Data Availability Statement

The raw data supporting the conclusions of this article are available upon reasonable request. Requests to access the datasets should be directed to Zhongrong Miao, Department of Interventional Neuroradiology, Beijing Tiantan Hospital, Capital Medical University, email: zhongrongm@163.com.

## Ethics Statement

The studies involving human participants were reviewed and approved by the Ethics Committees of Beijing Tiantan Hospital and all participating centers. The patients/participants provided their written informed consent to participate in this study.

## Author Contributions

YL, ZRM, JLN, YLD, FG, YJ, TTZ, XT, and BXJ conceived and designed the study. All authors assessed and diagnosed the patients. All authors were involved in the acquisition, analysis and interpretation of the data with JLN taking the primary role in statistical analysis. YL, JLN, YLD, FG, YJ, TTZ, XT, and BXJ drafted the manuscript. All authors were involved in revising the manuscript critically and gave final approval of the manuscript.

## Funding

This work was supported by the fifth 311 Project Scientific Research Funding Project in Taizhou (RCPY202004) and Taizhou Municipal Science and Technology Bureau (CN) (SSF20200086). The funder had no role in the study design, data collection, data analysis, data interpretation, writing of the report, decision to publish, or preparation of the manuscript.

## Conflict of Interest

The authors declare that the research was conducted in the absence of any commercial or financial relationships that could be construed as a potential conflict of interest.

## Publisher's Note

All claims expressed in this article are solely those of the authors and do not necessarily represent those of their affiliated organizations, or those of the publisher, the editors and the reviewers. Any product that may be evaluated in this article, or claim that may be made by its manufacturer, is not guaranteed or endorsed by the publisher.
