# Supplementary material for: Workflow Intervals and Outcomes of Endovascular Treatment for Acute Large-Vessel Occlusion During On-Vs. Off-hours in China: The ANGEL-ACT Registry
Source: Front Neurol. 2021 Dec 21;12:771803. doi: 10.3389/fneur.2021.771803 (PMC8724306; doi:10.3389/fneur.2021.771803)
Supplement: Supplementary file 1 [file Data_Sheet_1.PDF]

Supplementary Material

List of ANGEL-ACT study group:

Beijing Tiantan Hospital, Beijing, China: Zhongrong Miao, MD; Langfang Changzheng Hospital, Hebei, China: Liqiang Gui, MD; Liaocheng Third People’s Hospital, Shandong, China: Cunfeng Song, MD; The First People’s Hospital of Changzhou, Jiangsu, China: Ya Peng, MD; The Second Affiliated Hospital of Nanjing Medical University, Jiangsu, China: Jin Wu, MD; Fengrun District People’s Hospital of Tangshan City, Hebei , China: Shijun Zhao, MD; SiPing Central People’s Hospital, Jilin, China: Junfeng Zhao, MD; Yijishan Hospital of Wannan Medical College, Anhui, China: Zhiming Zhou, MD; The 2nd Affiliated Hospital of Harbin Medical University, Heilongjiang, China: Yongli Li, MD; The Central Hospital of Wuhan, Hubei, China: Ping Jing, MD; The First Hospital of Shijiazhuang, Hebei, China: Lei Yang, MD; Shenzhen Hospital of Southern Medical University, Guangdong, China: Yajie Liu, MD; The People’s Hospital of Longhua, Guangdong, China: Qingshi Zhao, MD; Jingjiang People’s Hospital, Jiangsu, China: Yan Liu, MD; The Third People’s Hospital of Hubei Province, Hubei, China: Xiaoxiang Peng, MD; The Second Affiliated Hospital of Guangzhou Medical University, Guangdong, China: Qingchun Gao, MD; Tianjin TEDA Hospital, Tianjin, China: Zaiyu Guo, MD; Zhangzhou Affiliated Hospital of Fujian Medical University, Fujian, China: Wenhuo Chen, MD; Taiyuan Central Hospital, Shanxi, China: Weirong Li, MD; The First Affiliated Hospital of Xinjiang Medical University, Xinjiang, China: Xiaojiang Cheng, MD; Affiliated Drum Tower Hospital of Nanjing University Medical School, Jiangsu, China: Yun Xu, MD; The First People’s Hospital of Wenling, Zhejiang, China: Yongqiang Zhang, MD; The Second Affiliated Hospital of Xi’an Jiaotong University, Shaanxi , China: Guilian Zhang, MD; The First People’s Hospital of Yulin, Guangxi, China: Yijiu Lu, MD; Zhenjiang First People’s Hospital, Jiangsu, China: Xinyu Lu, MD; Qitaihe Coal General Hospital Heilongjiang, China: Dengxiang Wang, MD; People’s Hospital of Tangshan City, Hebei , China: Yan Wang, MD; Affiliated Hospital of Guilin Medical University, Guangxi, China: Hao Li, MD; The Affiliated Hospital of Guizhou Medical University, Guizhou Province, China: Yang Hua, MD; The Affiliated Hospital of Xuzhou Medical University, Jiangsu, China: Deqin Geng, MD; Qingdao Central Hospital, ShanDong, China: Haicheng Yuan, MD; The Fourth People’s Hospital of Langfang City, Hebei , China: Hongwei Wang, MD; Beijing Daxing hospital, Beijing, China: Haihua Yang, MD; Weifang People’s Hospital,ShanDong, China: Zengwu Wang, MD; Luoyang General Hospital Affiliated to Zhengzhou University, Henan, China: Liping Wei, MD; Dongguan Kanghua Hospital, Guangdong, China: Xuancong Liufu, MD; Shunde Hospital of Southern Medical University, Guangdong, China: Xiangqun Shi, MD; Handan Central Hospital, Hebei, China: Juntao Li, MD; The 981 hospital of the Chinese People’s Liberation Army, Hebei , China: Wenwu Yang, MD; Linfen people’s Hospital, Shanxi, China: Wenji Jing, MD; Anshun people’s Hospital of Guizhou , China: Xiang Yong, MD; Changle People’s Hospital, Shandong, China: Leyuan Wang, MD; The Second People’s Hospital of Dongying, Shandong, China: Chunlei Li, MD; Tangshan Gongren hospital, HeBei , China: Yibin Cao, MD; PLA 985th Hospital of the Joint Logistics Support Force, Shanxi, China: Qingfeng Zhu, MD; Gaomi People’s Hospital, ShanDong, China: Peng Zhang, MD; Tongji Hospital, Tongji Medical College, Huazhong University of Science and Technology, Hubei, China: Xiang Luo, MD; Chongqing Sanxia Center Hospital, Chongqing, China: Shengli Chen, MD; Hospital of Traditional Chinese Medicine of Qiannan, Guizhou , China: WenWu Peng, MD; Guangdong Hospital of Chinese Medicine, Guangdong, China: Lixin Wang, MD; People’s hospital of Yangjiang， Guangdong, China: Xue Wen, MD; The Third Affiliated Hospital of CQMU, Chongqing, China: Shugui Shi, MD; General Hospital of The Yangtze River Shipping, Hubei, China: Wanming Wang, MD; First People’s Hospital of Bijie City,Guizhou, China: Wang Bo, MD; Suqian People’s Hospital of Nanjing Drum-Tower Hospital Group, Jiangsu, China: Pu Yuan, MD; Weifang TCM Hospital, Shandong, China: Dong Wang, MD; The Third Affiliated Hospital of Guangzhou Medical University, Guangdong, China: Haitao Guan, MD; Karamay Central hospital, Xinjiang, China: Wenbao Liang, MD; The third people’s Hospital of Xinjiang Uygur Autonomous Region, Xinjiang, China: Daliang Ma, MD; Wulanchabu City Central Hospital, Inner Mongolia, China: Long Chen, MD; Hospital of Xinjiang Production & Construction Corps , Xinjiang, China: Yan Xiao, MD; Jiaozuo Second people’s hospital, Henan, China: Xiangdong Xie, MD; 904th Hospital of Joint Logistic Support Force of PLA, Jiangsu, China: Zhonghua Shi, MD; Ganzhou People’s Hospital, Jiangxi, China: Xiangjun Zeng, MD; 967 Hospital of the Joint Logistics Support Force of PLA, Liaoning, China: Fanfan Su, MD; The Affiliated Hospital of Northwest University Xi’an No.3 Hospital, Shaanxi , China: MingZe Chang, MD; The Second Hospital of Liao Cheng, Shandong, China: Jijun Yin, MD; Jilin Province People’s Hospital, Jilin, China: Hongxia Sun, MD; People’s Hospital of Huanghua City, Hebei, China: Chong Li, MD; Shanghai Forth People’s Hospital, Shanghai, China: Yong Bi, MD; Wanbei Coal-electricity Group Gneral Hospital, Anhui, China: Gang Xie, MD; Shanghai Jiao Tong University Affiliated Sixth People’s Hospital, Shanghai, China: Yuwu Zhao, MD; Binzhou Medical University Hospital, Shandong, China: Chao Wang, MD; The 988 hospital of the people’s

liberation army, Henan , China: Peng Zhang, MD; Linyi People’s Hospital, Shandong, China: Xianjun Wang, MD; Yingkou City Central Hospital, Liaoning, China: Dongqun Li, MD; Yantaishan Hospital, Shandong, China: Hui Liang, MD; Mianyang Central hospital, Sichuan, China: Zhonglun Chen, MD; Chengdu Fifth People’s Hospital, Sichuan, China: Yan Wang, MD; Hengshui Fifth Hospital of Heng shui City, HeBei, China: Yu Xin, Wang, MD; Second Hospital of Dalian Medical University, Liaoning, China: Lin Yin, MD; Boai Hospital of Zhongshan, Guangdong, China: HongKai Qiu, MD; The First People’s Hospital of Yibin, Sichuan , China: Jun Wei, MD; Shanxi provincial people’s hospital, Shanxi, China: Yaxuan Sun, MD; Shandong Provincial Third Hospital, Cheeloo College of Medicine, Shandong University, Shandong, China: Xiaoya Feng, MD; Chuxiong State People’s Hospital, Chuxiong, Yunnan, China: Weihua Wu, MD; The Fourth Affiliated Hospital of China Medical University, Liaoning, China: Lianbo Gao, MD; Taihe Hospital, Shiyan, Hubei , China: Zhibing Ai, MD; Qingdao Municipal Hospital，Shandong, China: Tan Lan, MD; The First People’s Hospital of Yunnan Province, Yunnan, China: Li Ding, MD; The NO.2 People’s Hospital of Lanzhou. Gansu, China: Qilong Liang, MD; Taizhou First People’s Hospital, Zhejiang, China: Zhimin Wang, MD; Hunan Provincial People’s Hospital, Hunan, China: Jianwen Yang, MD; First People’s Hospital of Changde City, Hunan, China: Ping Xu, MD; Zhejiang Yuyao People’s Hospital, Zhejiang, China: Wei Dong, MD; AideBao Hospital, HeBei , China: Quanle Zheng, MD; The First Hospital of Fangshan District, Beijing, China: Zhenyun Zhu, MD; Tianjin Xiqing Hospital, Tianjin, China: Liyue Zhao, MD; People’s Hospital of Zunhua, Hebei, China: Qingbo Meng, MD; Xingtai Third Hospital, Hebei, China: Yuqing Wei, MD; Qingyuan People’s Hospital, Guangdong , China: Xianglin Chen, MD; Fengcheng City Central Hospital，Liaoning, China: Wei Wang, MD; People’s Hospital of Hejian City, Hebei , China: Dong Sun, MD; Hangzhou Third People’s Hospital, Zhejiang, China: Yongxing Yan, MD; Xiangtan Central Hospital, Hunan, China: Guangxiong Yuan, MD; People’s Hospital of Nanpi Country, Hebei , China: Yadong Yang, MD; Liuzhou Railway Central Hospital, Guangxi, China: Jianfeng Zhou, MD; Maoming People’s Hospital, Guangdong, China: Zhi Yang, MD; Tongde Hospital of Zhejiang Province, Zhejiang, China: Zhenzhong Zhang, MD; The First Affiliated Hospital of Jinzhou Medical University, Liaoning, China: Ning Guan, MD; Xishan coal electricity group worker general hospital, Shaanxi, China: Huihong Wang, MD.

Study protocol

NIH U.S. National Library of Medicine
ClinicalTrials.gov

Endovascular Treatment Key Technique and Emergency Work Flow Improvement of Acute Ischemic Stroke (ANGEL-ACT)

The safety and scientific validity of this study is the responsibility of the study sponsor and investigators. Listing a study does not mean it has been evaluated by the U.S. Federal Government. Read our disclaimer for details.

ClinicalTrials.gov Identifier: NCT03370939

Recruitment Status : Completed
First Posted : December 13, 2017
Last Update Posted : June 1, 2020

Sponsor:
Beijing Tiantan Hospital

Information provided by (Responsible Party):
Zhongrong Miao, Beijing Tiantan Hospital

Study Description

Go to

Brief Summary:

Endovascular thrombectomy (EVT) is effective and safe for acute ischemic stroke (AIS) caused by large vessel occlusion (LVO) in major clinical trials. Whether the benefit of EVT in randomized trials could be generalized to clinical practice, especially in developing countries, remains unknown. The prospective Chinese ANGEL-ACT Registry (Endovascular Treatment Key Technique and Emergency Work Flow Improvement of Acute Ischemic Stroke) was established to evaluate the utilization, and subsequent outcomes of EVT treated AIS patients. This study is a multi-center, prospective registry study initiated by researchers, funded by National Key R&D Program of China. A total of 2,000 patients with acute ischemic stroke will undergo endovascular treatment. The hypothesis was that favorable outcomes from clinical trials could be achieved in clinical practice in China.

Condition or disease
Intracranial Artery Occlusion With Infarction (Disorder)

Study Design

Go to

Study Type :
Observational [Patient Registry]

Actual Enrollment :
2004 participants

Observational Model:
Case-Only

Time Perspective:
Prospective

Target Follow-Up Duration:
90 Days

Official Title:
Endovascular Treatment Key Technique and Emergency Work Flow Improvement of Acute Ischemic stroke-a Prospective Multicenter Registry Study

Actual Study Start Date :
November 11, 2017

Actual Primary Completion Date :
July 7, 2019

Actual Study Completion Date :
July 7, 2019

Groups and Cohorts

Go to

Outcome Measures

Go to

Primary Outcome Measures :

1. Functional independence at 90 days (modified Rankin Scale of 0-2) [Time Frame: 90±7 days after enrollment]

   The range of modified Rankin Scale was from 0 to 6. 0-No symptoms;1-No significant disability;2-Slight disability;3-Moderate disability;4-Moderately severe disability;5-Severe disability;6 -Dead.A higher score indicates worse a outcome.

2. Symptomatic intracranial hemorrhage (sICH) within 12-36 hours after the procedure [Time Frame: 12-36 hours after the procedure]

   Heidelberg Bleeding Classification): new intracranial hemorrhage detected by brain imaging associated with ≥4 points total National Institutes of Health Stroke Scale (NIHSS), ≥2 points in one NIHSS category, leading to intubation/ hemicraniectomy/ EVD placement or other major medical/surgical intervention, or absence of alternative explanation for deterioration

3. Time from symptom onset to recanalization [Time Frame: The end of the procedure]

Secondary Outcome Measures :

1. Recanalization rate at the end of the procedure [Time Frame: at the end of the procedure]

   mTICI score 2b-3

2. Recanalization rate after the first attempt [Time Frame: At baseline, during the procedure, after the first attempt of endovascular treatment]

   mTICI score 2b-3

3. Changes in NIHSS score immediately after the procedure [Time Frame: within 2 hours after the procedure]

   difference between NIHSS score immediately after the procedure and baseline

4. Changes in NIHSS score 24 hours after the procedure [Time Frame: 24 hours after the procedure]

   difference between NIHSS score 24 hours after the procedure and baseline

5. Changes in NIHSS score 7 days after the procedure or at discharge [Time Frame: 7 days after the procedure or discharge]

   difference between NIHSS score 7 days after the procedure or discharge and baseline

6. EQ-5D 90 days after the procedure [Time Frame: 90±7 days after the procedure]

EQ-5D is a standardized instrument for measuring generic health status. Rated level can be coded as a number 1, 2, or 3, which indicates having no problems for 1, having some problems for 2, and having extreme problems for 3. As a result, a person's health status can be defined by a 5-digit number, ranging from 11111 (having no problems in all dimensions) to 33333 (having extreme problems in all dimensions).A higher score indicates a better outcome.

7. Barthel index (BI) 90 days after the procedure [Time Frame: 90±7 days after the procedure]

   The BI has a score of 0-100. A higher score indicates a better outcome.

8. Parenchymal hematoma (PH2) [Time Frame: 12-36 hours after the procedure]

   PH2 is defined as hematoma in >30% of infarct area

9. Any intracranial hemorrhage on imaging [Time Frame: 12-36 hours after the procedure]
10. All-cause mortality within 90 days [Time Frame: 90±7 days after the procedure]
11. Time from onset to arrival [Time Frame: At baseline, after arrival at the hospital]
12. Time from arrival to imaging [Time Frame: At baseline, after taking any brain imaging]
13. Time from imaging to puncture [Time Frame: At baseline, during the procedure, after successful groin puncture]
14. Time from puncture to recanalization [Time Frame: At baseline, during the procedure, after successful recanalization]

Eligibility Criteria

Go to

Information from the National Library of Medicine

NIH NLM

Choosing to participate in a study is an important personal decision. Talk with your doctor and family members or friends about deciding to join a study. To learn more about this study, you or your doctor may contact the study research staff using the contacts provided below. For general information, Learn About Clinical Studies.

Ages Eligible for Study:
18 Years and older (Adult, Older Adult)

Sexes Eligible for Study:
All

Accepts Healthy Volunteers:
No

Sampling Method:

Non-Probability Sample

Study Population

Patients with acute ischemic stroke caused by large vessel occlusion and receiving endovascular treatment.

Criteria

Inclusion criteria

1. Age ≥ 18 years old;
2. Diagnosis of acute ischemic stroke;
3. Imaging confirmed intracranial large artery occlusion (LVO): intracranial internal carotid artery (ICA T/L), middle cerebral artery (MCA M1/M2), anterior cerebral artery (ACA A1/A2), basilar artery (BA), vertebral artery (VA V4), and posterior cerebral artery (PCA P1);
4. Initiation of any type of endovascular treatment (EVT), including intra-arterial thrombolysis, mechanical thrombectomy, angioplasty, and stenting;
5. The patient or the patient's legal representative is able and willing to sign the informed consent.

Exclusion criteria

1. Isolated cervical ICA or VA occlusion;
2. No evidence of LVO on DSA.

Contacts and Locations

Go to

Information from the National Library of Medicine

NIH NLM

To learn more about this study, you or your doctor may contact the study research staff using the contact information provided by the sponsor.

Please refer to this study by its ClinicalTrials.gov identifier (NCT number): ***NCT03370939***

Locations

China, Beijing

Beijing Tiantan Hospital
Beijing, Beijing, China, 100010

Sponsors and Collaborators

Beijing Tiantan Hospital

Investigators

Principal Investigator: Zhongrong Miao, PhD Capital Medical University, Beijing Tiantan Hospital

More Information

Go to

Additional Information:

Publications automatically indexed to this study by ClinicalTrials.gov Identifier (NCT Number):

Tong X, Wang Y, Bauer CT, Jia B, Zhang X, Huo X, Luo G, Wang A, Ma N, Gao F, Mo D, Song L, Sun X, Liu L, Deng Y, Li X, Wang B, Ma G, Wang Y, Ren Z, Miao Z. Current status of aspiration thrombectomy for acute stroke patients in China: data from ANGEL-ACT Registry. Ther Adv Neurol Disord. 2021 Apr 12;14:17562864211007715. doi: 10.1177/17562864211007715. eCollection 2021.

Tong X, Wang Y, Fiehler J, Bauer CT, Jia B, Zhang X, Huo X, Luo G, Wang A, Pan Y, Ma N, Gao F, Mo D, Song L, Sun X, Liu L, Deng Y, Li X, Wang B, Ma G, Wang Y, Ren Z, Miao Z; ANGEL-ACT Study Group. Thrombectomy Versus Combined Thrombolysis and Thrombectomy in Patients With Acute Stroke: A Matched-Control Study. Stroke. 2021 May;52(5):1589-1600. doi: 10.1161/STROKEAHA.120.031599. Epub 2021 Mar 4.

Jia B, Ren Z, Mokin M, Burgin WS, Bauer CT, Fiehler J, Mo D, Ma N, Gao F, Huo X, Luo G, Wang A, Pan Y, Song L, Sun X, Zhang X, Gui L, Song C, Peng Y, Wu J, Zhao S, Zhao J, Zhou Z, Li Y, Jing P, Yang L, Liu Y, Zhao Q, Liu Y, Peng X, Gao Q, Guo Z, Chen W, Li W, Cheng X, Xu Y, Zhang Y, Zhang G, Lu Y, Lu X, Wang D, Wang Y, Li H, Ling L, Peng G, Zhang J, Zhang K, Li S, Qi Z, Xu H, Tong X, Ma G, Liu R, Guo X, Deng Y, Leng X, Leung TW, Liebeskind DS, Wang Y, Wang Y, Miao Z; ANGEL-ACT Study Group†. Current Status of Endovascular Treatment for Acute Large Vessel Occlusion in China: A Real-World Nationwide Registry. Stroke. 2021 Apr;52(4):1203-1212. doi: 10.1161/STROKEAHA.120.031869. Epub 2021 Feb 18.

Responsible Party:
Zhongrong Miao, Director, Department of interventional neurology, Beijing Tiantan Hospital

ClinicalTrials.gov Identifier:
NCT03370939 History of Changes

Other Study ID Numbers:
2016YFC1301501-1

First Posted:
December 13, 2017 Key Record Dates

Last Update Posted:
June 1, 2020

Last Verified:
May 2020

Studies a U.S. FDA-regulated Drug Product:

No

Studies a U.S. FDA-regulated Device Product:

No

Keywords provided by Zhongrong Miao, Beijing Tiantan Hospital:

ischemic stroke

endovascular treatment

registry

Additional relevant MeSH terms:

Infarction

Emergencies

Ischemia

Pathologic Processes

Necrosis

Disease Attributes
